# Biodegradation of Plastics Induced by Marine Organisms: Future Perspectives for Bioremediation Approaches

**DOI:** 10.3390/polym15122673

**Published:** 2023-06-14

**Authors:** Thomas Viel, Loredana Manfra, Valerio Zupo, Giovanni Libralato, Mariacristina Cocca, Maria Costantini

**Affiliations:** 1 Department of Ecosustainable Marine Biotechnology, Stazione Zoologica Anton Dohrn, Villa Comunale, 80121 Napoli, Italy; thomas.viel@szn.it (T.V.); loredana.manfra@isprambiente.it (L.M.); giovanni.libralato@unina.it (G.L.); maria.costantini@szn.it (M.C.); 2 Institute for Polymers, Composites and Biomaterials, National Research Council of Italy, Via Campi Flegri, 34, 80078 Pozzuoli, Italy; 3 Department of Biology, University of Naples Federico II, Via Cinthia 26, 80126 Napoli, Italy; 4 Institute for Environmental Protection and Research (ISPRA), Via Vitaliano Brancati 48, 00144 Rome, Italy; 5 Stazione Zoologica, Ecosustainable Biotechnology Department, Ischia Marine Centre, Via Buonocore 42, 80077 Ischia, Italy; valerio.zupo@szn.it

**Keywords:** biodegradation, plastics, marine organisms

## Abstract

Plastic pollution is a distinctive element of the globalized world. In fact, since the 1970s the expansion and use of plastics, particularly in the consumer and commercial sectors, has given this material a permanent place in our lives. The increasing use of plastic products and the wrong management of end-of-life plastic products have contributed to increasing environmental pollution, with negative impacts on our ecosystems and the ecological functions of natural habitats. Nowadays, plastic pollution is pervasive in all environmental compartments. As aquatic environments are the dumping points for poorly managed plastics, biofouling and biodegradation have been proposed as promising approaches for plastic bioremediation. Known for the high stability of plastics in the marine environment, this represents a very important issue to preserve marine biodiversity. In this review, we have summarized the main cases reported in the literature on the degradation of plastics by bacteria, fungi, and microalgae and the degradation mechanisms involved, to highlight the potential of bioremediation approaches to reduce macro and microplastic pollution.

## 1. Introduction

Global plastic production reached enormous values, with 367 million tons (Mt) of plastics produced in 2020, not including polyester, polyamide, and polyacrylic fibers productions [1,2]. Plastics, in general, are the most commonly used material in the world for different sectors, such as automotive, packaging, pharmaceutics, construction, and design, since they are designed to have excellent properties for specific applications [3]. Unfortunately, the recycling share of this waste is still low and only one-third of it ends up in recycling centers in Europe [1]. Furthermore, some of it will still end up in the environment and it was estimated that between 10 and 20 Mt of plastics end up in the oceans each year and that, by 2050, the amount of plastics might exceed the world fish stocks [4,5]. Currently, plastic waste is found everywhere on land, as well as deep in the oceans [6]. The most common plastics found in the aquatic environments are commonly used polymers such as high-density polyethylene (HDPE), low-density polyethylene (LDPE), polyvinyl chloride (PVC), polystyrene (PS), polypropylene (PP) and polyethylene terephthalate (PET) [7]. These materials can accumulate in the different environmental compartments, particularly in the aquatic ones, leading to the formation, during their degradation, of meso-, micro- or nano-plastics, generally defined as secondary particles [8]. In addition to these, primary microplastics considered here as particles produced in a sub-millimetric size for a specific application and particles originating from large plastic items (during their manufacturing, use, or maintenance) strongly contribute to increase the magnitude of pollution [9]. This poses significant problems for global ecosystems and claims for awareness [10]. These plastics can have significant ecotoxicological effects in the environment and, furthermore, depending on the type of plastic, their effects can be totally different. Consequently, it is important to carefully monitor each type of plastic [11,12,13]. Recently, many research activities have been finalized on the development of reliable and sustainable technological strategies to reduce plastic pollution; some of them are based on the improvement of polymer degradation.

Polymer degradation depends on several factors and involves chemical, physical, and biological reactions resulting in bond scissions and subsequent changes in material properties and appearance. Depending upon the cause, polymer degradations have been classified as photo-oxidative degradation, thermal degradation, ozone-induced degradation, mechanochemical degradation, catalytic degradation, and biodegradation [14]. Typically, chemical degradation in the environment involves complex mechanisms such as hydrolysis or oxidation, or both, which can be accelerated by microbial action, heat, light, or their combinations [15]. The degradation behavior of several polymers has been studied and reported in the literature, such as among non-biodegradable polymers PE, PET, PVC, PP, and PS, as and among biodegradable polymers polylactic acid (PLA), poly(ɛ-caprolactone) (PCL) [15]. PET, for example, represents about 12% of global solid waste, and a rapid enzymatic depolymerization can help a circular carbon economy for PET with a conversion/valorization into other products [13,16]. 

This review is mainly focused on the degradation of plastics by marine organisms to investigate how different polymers behave in the marine environment and to highlight the potential of bioremediation approaches to reduce microplastic pollution. Therefore, it is important to find strategies to reduce the presence of plastic waste in ecosystems and particularly in marine ecosystems. In this scenario, this review summarizes and analyzes recent studies concerning the mechanisms and processes of plastic degradation by organisms, particularly marine organisms, with a view to bioremediation approaches. Plastic degradation by organisms occurs through either abiotic or biotic processes [15].

Three main degradation mechanisms for plastics occur in the marine environment: (i) colonization of plastics via microorganisms forming a biofilm on the polymer surface that induce biodegradation via surface erosion; (ii) abiotic hydrolysis of functional groups (esters, carbonates, and amides), accounts for the reduction of molecular weight, which could be favored by the presence of hydroxide ions; and (iii) exposure to UV light and oxygen causes photodegradation and results in a reduction of molecular weight and cracking of the material. Several techniques can be used to follow the degradation of polymeric materials. An easy quantification of the extent of polymer degradation can be obtained by evaluating weight changes, by determining the mass loss. However, the process of degradation cannot be studied and interpreted alone. It must be combined with other analytical methods such as the evolution of CO_2_. Under anaerobic conditions, soluble carbon compounds are metabolized by methanogens or sulfate reducers, producing CH_4_ and CO_2_, respectively. The rate of biodegradation can be monitored by measuring the amount of CO_2_ released. Gel permeation chromatography (GPC) is useful to determine changes in polymer molecular weight during degradation, since biotic and abiotic degradation can lead to chain scissions, with the formation of low molecular weight chains and oligomers. Molecular analysis of polymer by nuclear magnetic resonance (NMR) and Fourier transform infrared (FTIR) spectroscopies can also be used to determine the occurrence of oxidation/hydrolysis processes during degradation, as well as to detect the presence of functional groups of degraded products in the polymers. Moreover, degradation can be followed by determining changes in physical and functional properties. As a matter of fact, polar functional groups can change polymer hydrophobicity/hydrophilicity during the degradation. This process can be evaluated by water or solvent contact angle measurements. Finally, mechanical and dynamic mechanical analyses of the materials can be used to determine the occurrence of degradation. Changes in glass and phase transitions of polymers can be evaluated via differential scanning calorimetry (DSC), while the degradation effect on material thermal stability can be evaluated using thermogravimetric analysis (TGA). Morphological analysis using scanning electron microscopy (SEM) allows us to observe degradation phenomena on polymer surfaces. All these methods permit us to determine and follow the degradation of a polymer, according to the triggers to which it is exposed.

This review aims at providing a state of the art in the knowledge of degradation of polymers performed by marine species and the method used to assess the organism’s ability to induce degradation and to outline the mechanisms used by these species to achieve plastic degradation. To our knowledge, few studies summarized in a single work the three major groups that are found in the literature involved in the above-described processes: bacteria, fungi, and microalgae. An important point highlighted in this work will also be the challenge of using marine microorganisms as a real opportunity for the bioremediation of the marine environment from plastics through marine organisms able to degrade them. 

## 2. Degradation by Bacteria

According to the ASTM standard D-5488-94d, biodegradation is defined as “a process which is capable of decomposition of materials into carbon dioxide, methane, water, inorganic compounds, or biomass in which the predominant mechanism is the enzymatic action of microorganisms, which can be measured by standard tests, in a specified period of time, reflecting available disposal conditions”. Plastic biodegradation by bacteria occurs in aerobic and anaerobic conditions [17,18,19], and their attachment to plastic surfaces is possible thanks to the presence of the matrix of extracellular polymeric substances [20]. The molecular mechanism involved in biofilm development obviously depends on the microbial species. Under aerobic conditions, oxygen is used by the bacteria during degradation to convert polymers into CO_2_ and H_2_O; differently, under anaerobic conditions bacteria use nitrate, sulfate, iron, manganese, and carbon dioxide as electron acceptors to break down the polymers into smaller compounds CO_2_, CH_4_, and H_2_O [21]. These chemical processes can be represented by the following reaction schemes (Equations (1) and (2)) [22].

Aerobic conditions:

(1) $$C_{\mathrm{polymer}}+O_{2}\to \;{\mathrm{CO}}_{2}+H_{2}O+{\;C}_{\mathrm{residue}}+{\;C}_{\mathrm{biomass}}+\;\mathrm{salts}$$

Anaerobic conditions:

(2) $$C_{\mathrm{polymer}}\to \;{\mathrm{CO}}_{2}+H_{2}O+{\;\mathrm{CH}}_{4}+C_{\mathrm{residue}}+{\;C}_{\mathrm{biomass}}+\;\mathrm{salts}$$

**Figure 1:**
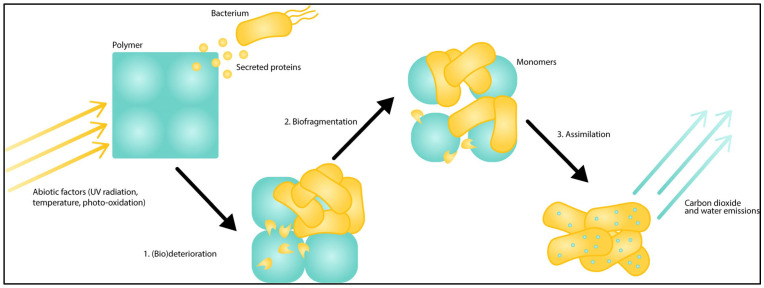
Schematic illustration of plastic biodegradation as a complex, step-wise pathway. First, during biodeterioration (1), abiotic and biotic factors, such as light and secreted hydrophobins, modify the polymer surface and increase its surface area. Next, during biofragmentation (2), organisms secrete enzymes that break the polymer down into its monomer components. Finally, during assimilation (3), the organism metabolizes the monomers into biomass, carbon dioxide, and water [27]. Copyright © 2019 Sheth, Kwartler, Schmaltz, Hoskinson, Martz, Dunphy-Daly, Schultz, Read, Eward and Somarelli. An open-access article distributed under the terms of the Creative Commons Attribution License (CC BY).

When complete mineralization of polymers occurs, no residues remain, i.e., the polymer is completely converted into gases and water. Bacteria use polymers as a source of energy by secreting extracellular enzymes. Polymers are degraded by enzymes outside the bacterial cells [23]. The degradability of plastics depends on many factors, including environmental factors, such as pH, temperature, salinity [24], and polymer properties. Indeed, Gu (2003) reported that the degradability of polymers depends on molecular weight, crystallinity, and physical form (molecular organization) [18]. In general, an increase in the molecular weight of the polymer will result in less degradation by microorganisms. Moreover, it has been shown that bacterial communities colonizing plastics are diverse and that their composition depends on the nature of the material, but also on its geographical location. In fact, different bacterial communities have been found to develop on floating plastics and plastics present in the sediments of the same geographical area [25]. The nature and catalytic activities of the enzymes involved in the degradation process vary between species; different enzymes are known to be more or less efficient, depending on the type of polymer. For example, for the degradation of PCL, the bacteria *Rhizopus delemar* and *Clostridium botulinum* use lipase as a degradation enzyme [26]. The effects of some marine bacteria are summarized in Table 1, showing plastic degradation capabilities as described in the literature. A general process of plastic degradation by bacteria can be nevertheless presented (Figure 1).

The first step is the attachment and colonization by the bacteria on a plastic surface. Bacteria must change the hydrophobicity of the polymer by producing surfactants in order to adhere directly to the material [28]. This step is most effective when bacteria are deprived of other carbon sources. Indeed, when the polymer is the only source of carbon, hydrophobic interactions are enhanced and the adhesive behavior of the bacteria is increased, causing them to start the degradation process more rapidly [29]. In the marine environment, this process has been shown to occur rapidly, due to significant changes in the physical–chemical properties (i.e., surface hydrophobicity and buoyancy) of polymers [30]. Furthermore, the degradability of polymers is likely to be improved by supplementing polymers with additives, which affect their thermal sensitivity and UV absorption capacity [31]. Indeed, the use of benzophenone as an additive would play an important role during photodegradation [32].

Following this attachment and colonization by bacteria, plastics start to depolymerize due to the enzyme action. This process involves hydrolysis, which represents the most important reaction in the degradation induced by bacteria [33]. In fact, in different microorganisms intracellular or extracellular depolymerases can degrade polymers. In the case of intracellular degradation, an endogenous carbon reservoir is involved through the accumulation of microbes themselves, whereas when extracellular degradation occurs, an exogenous carbon source is requested, not necessarily linked to the accumulation of microorganisms. In that way, enzymes from microorganisms divided polymers into short chains or smaller molecules able to pass through membranes using the mechanism known as depolymerization. In turn, these short molecules are subjected to the mineralization of end products, such as CO_2_, H_2_O or CH_4_, and utilized as sources of carbon and energy [18]. Many enzymes directly related to the degradation of plastics have already been isolated and reported in the literature. Lipases from *Rhizopus arrhizus*, *R. delemar*, *Achromobacter* sp., and *Candida cylindracea* have shown activities on PEA and PCL Proteinase K secreted by *Tritirachium album* show great efficiency in the degradation of PLA, as well as α-chymotrypsin [34]. Regarding the polyethylene polymer, the bacteria present in this material possess a gene, the alkB gene, coding for alkane 1-monooxygenase [35]. This is considered one of the key players in the degradation of polyethylene [28]. 

*Ideonella sakaiensis* can use PET as its main energy and carbon source. In particular, this bacterial strain produces two enzymes (ISF6-4831 and ISF6-0224) able to hydrolyze PET and PE [33]. PCL can be degraded by lipases and esterases with a degradation rate mainly affected by PCL molecular weight and degree of crystallinity [36]. A cutinase-like enzyme (an enzyme that was originally described for the hydrolysis of cutin, a natural polyester constituting plant cuticle), has been identified to hydrolyze polyamide-epicloroidrina dipentyl phthalate to phthalate [37]. Sekiguchi et al. studied PCL, polyhydroxybutyrate (PHB), and polybutylene succinate (PBS) degradation in the deep sea at Rausu, Toyama, and Kume, Japan [38]. After 12 months of immersion, PCL and PHB fibers became brittle or completely degraded. Five PCL-degrading bacteria were isolated by applying phylogenetic analysis using 16S rRNA gene sequencing, belonging to the genera *Pseudomonas*, *Alcanivorax*, and *Tenacibaculum*. All these bacteria were able to degrade PCL fibers in vitro. Furthermore, it was demonstrated that these strains preferred conditions of low temperature, ranging from 4 to 10 °C, and high hydrostatic pressure for the degradation. In addition, in 2013 Harshvardhan and Jha isolated sixty marine bacteria from pelagic waters exhibiting abilities to degrade low-density polyethylene (LDPE) when it represents the unique carbon source [39]. Three isolates were Gram-positive and identified as *Kocuria palustris* M16, *Bacillus pumilus* M27, and *Bacillus subtilis* H1584 based on 16S rRNA gene sequence homology. LDPE lost 1%, 1.5%, and 1.75% of weight after 30 days of incubation with the M16, M27, and H1584, respectively. Raghul et al. reported that marine bacteria, *Vibrio alginolyticus* and *Vibrio parahemolyticus*, isolated from sediments, are able to degrade plastic films made with polyvinyl alcohol and LDPE after 15 weeks under shake flask conditions at 120 rpm at 37 °C [40]. The study indicated the potential of marine benthic bacteria with novel enzymes and unique characteristics for application in bioremediation and plastic waste degradation. In addition, other bacteria have been identified to play a role in the degradation of linear low-density polyethylene (LLDPE) [28]. In the degradation experiment carried out in the microcosm, *Lysinibacillus* sp. and *Salinibacterium* sp. were isolated and identified. Degradation studies of PVC, LDPE, and HDPE permitted us to define the role of the marine bacterial strain AIIW2 (with a similarity of 97.4% with *Bacillus* species, identified by using the 16S rRNA gene sequence) growth on the plastic surface [41]. The degradation of plastics was followed by measuring weight loss after 90 days of incubation with the bacterial strain; 0.26 ± 0.02, 0.96 ± 0.02, and 1.0 ± 0.01% of weight loss for PVC, LDPE, and HDPE films, respectively, was obtained after 90 days of incubation. The results of this study revealed the ability of marine bacterial strains to colonize plastic films and degrade the polymer structure. Moreover, Mohanrasu et al. reported the marine bacterium *Brevibacillus borstelensis,* identified by 16S RNA sequencing, found in the coastal regions from northeastern to southern Tamilnadu in India, exhibits a degrading capacity for HDPE. HDPE degradation was followed by confocal laser scanning microscopy (CLSM), Fourier transform infrared spectroscopy (FTIR), and scanning electron microscopy (SEM) [42].

Auta et al. studying polypropylene, demonstrated the degradation mechanism of marine bacteria from mangrove sediments [43]. *Rhodococcus* sp. strain 36 and *Bacillus* sp. were able to use the PP for growth, as confirmed by the reduction of the mass of the PP. In fact, they found that the weight loss was 6.4% for *Rhodococcus* sp. strain 36 and 4.0% for *Bacillus* sp. strain 27 after 40 days of incubation. Balasubramanian et al. were interested in HDPE-degrading bacteria [44]. Fifteen bacteria (GMB1-GMB15 strains) were isolated by the enrichment technique. GMB5 and GMB7 strains were selected on the basis of their efficiency in degrading HDPE and identified as *Arthrobacter* sp. and *Pseudomonas* sp., respectively. The estimated weight loss of HDPE after 30 days of incubation was about 12% for *Arthrobacter* sp. and 15% for *Pseudomonas* sp. Urbanek et al. studied bacteria from cold marine habitats, demonstrating the ability of *Pseudomonas* sp., *Rhodococcus* sp., and *Clonostachys rosea* to degrade poly(butylene succinate-co-adipate) (PBSA), poly(butylene succinate) (PBS), PCL, and PLA [31]. Not often cited in the literature, Sudhakar et al. investigated the case of nylon 66 (N66) and nylon 6 (N6) and their degradation by marine bacillus such as *Bacillus cereus*, *Bacillus sphericus*, *Vibrio furnisii* and *Brevundimonas vesicularis* [27]. They found that these bacteria decreased the average molecular weight of N66 and N6 by 42% and 31% and the weight of N66 and N6 decreased by 7% and 2%, respectively. Furthermore, many bacteria showed degradation activity towards PE: *Muricauda* sp., *Pelomonas* sp., *Sphingomonas* sp., *Acinetopbacter* sp., *Staphylococcus epidermidis*, and *Thalassospira* sp. [45].

**Table 1:** List of bacterial species reported in the literature responsible for plastic degradation, types of plastics involved, type of degradation detected, and localization of the activity, according to literature references (reported in the last column).

Bacteria	Plastic Type	Observation of Degradations	Localization	Reference
Genus *Pseudomonas*, *Alcanivorax*, *Tenacibaculum*	PCL, poly(b-hydroxybutyrate/valerate) (PHB/V), polybutylene succinate (PBS)	Morphological observation (SEM); tensile strength	Deep sea	[38]
*Pseudomonas* sp., *Rhodococcus* sp., *Clonostachys rosea*	PBSA, polybutylene succinate-co-adipate (PBS), PCL, PLA	Morphological observation (SEM)	Arctic region	[31]
*Kocuria palustris Bacillus pumilus*, *Bacillus subtilis*	LDPE	Weight loss; FT-IR observation	Pelagic Seawater	[39]
*Vibrio alginolyticus*, *Vibrio parahemolyticus*	Polyvinyl alcohol linear low-density polyethylene (PVA-LLDPE)	Morphological observation (SEM); breaking strength	Seawater	[40]
Genus *Bacillus*	PVC, LDPE, HDPE	Weight loss; FT-IR observation; morphological observation (SEM)	Seawater	[41]
*Brevibacillus borstelensis*	HDPE	FT-IR observation; morphological observation (SEM)	Seawater	[42]
*Pseudomonas* sp., *Arthrobacter* sp.	HDPE	Weight loss; FT-IR observation	Marine ecosystem	[44]
*Lysinibacillus* sp., *Salinibacterium* sp.	PE	FT-IR observation; morphological observation (SEM)	Surface seawater	[28]
*Rhodococcus* sp., *Bacillus* sp.	PP	Weight loss; FT-IR observation; morphological observation (SEM)	Mangrove sediment	[43]
*Bacillus cereus*, *Bacillus sphericus*, *Vibrio furnisii*, *Brevundimonas vesicularis*	N6, N66	Weight loss; FT-IR observation; DSC; epi-fluorescence microscopy	Marine ecosystem	[27]
*Muricauda* sp., *Pelomonas* sp., *Sphingomonas* sp., *Acinetopbacter* sp., *Staphylococcus epidermidis*, *Thalassospira* sp.	Polyester		Marine ecosystem	[45]
*Thalassospira povalilytica*	PVA		Marine ecosystem	[46]

## 3. Degradation by Marine Fungi

The general process of polymer degradation by marine fungi is similar to the process operated by bacteria and algae. In fact, the fungi action proceeds through attachment, colonization, and further degradation of the polymer. HDPE films were degraded by two marine fungi, *Aspergillus tubingensis* and *Aspergillus flavus* [47]. The morphology of the polymer surface was strongly affected by the action of fungi with the appearance of cracks. The polymers that were not affected by the fungi kept their surface smooth and intact even after 30 days, while the presence and action of enzymes causes the appearance of cracks, thus indicating degradation by the colonizing fungi. Fungi, similar to bacteria, grow and use polymer as their sole carbon source, thus, degrading it [48] (Ameen et al., 2015). In fungal attachment, an important role is played by laccases, which are multicopper oxidases that oxidize a wide variety of substrates, including polymers [49], as well as lignin, PE, and PVC. The esterases, such as cutinases and lipases, were very useful in degradation of PET and polyurethane (PUR), and on this last one also proteases and ureases showed very good results [50]. Mainly basidiomycetes and ascomycetes have good efficiency in plastic biodegradation under laboratory conditions.

Various other enzymes have been reported in the literature for their degradation activity on polymeric substrates; among them, three cutinases (from *Humilica insolens*, *Pseudomonas medicine*, and *Fusarium solani*) show degradation ability on PET [51]. Wang et al. cultivated various species of fungi and thus extracted and isolated several hydrolytic enzymes, including amylases, glucanases, xylanases, pectinases, and lipases, able to hydrolyze various polymers of algae [52]. Zang et al. found two laccase genes, AFLA_006190 and AFLA_053930, which exhibited high expression during the degradation process of HDPE [53]. All these data showed a large variety of enzymes used during the degradation process. In fact, recently it was demonstrated that the enzymes are not used alone, but synergistically to increase efficiency [54]. Indeed, in *Peniophora* sp., the presence of at least eight genes has been highlighted; they can encode ten different laccases, representing a valuable source with a high potential for the exploitation of this fungus in bioremediation purposes [55].

Marine fungal species with polymer degradation ability are listed in Table 2. Devi et al. identified various fungal strains able to degrade HDPE [47]. HDPE degradation induced by two fungal strains, coded as VRKPT1 and VRKPT2, was followed by weight loss determination and FTIR analysis. The isolated fungi were identified as *Aspergillus tubingensis* VRKPT1 and *Aspergillus flavus* VRKPT2. Biofilm formation observed by epifluorescence microscopy showed the activity of the fungal strains even after one month of incubation.

Alshehrei isolated ten fungal strains from the water of the Red Sea (Jeddah, Saudi Arabia) [56]. The selected fungi, related to *Aspergillus* and *Penicillium*, showed the ability to degrade LDPE film and powder. *Penicillium* sp. showed the highest PE weight loss reduction (43.4%). Ameen et al., studying LDPE, identified forty-five fungal isolates belonging to thirteen genera from tidal water, floating debris, and sediments collected from mangrove stands on the Red Sea coast in Saudi Arabia [48]. Six of these isolates and their consortia were found to grow in association with LDPE film during in vitro experiments in the absence of dextrose or another carbon source: *Aspergillus caespitosus*, *Phialophora alba*, *Paecilomyces variotii*, *Aspergillus terreus*, *Alternaria alternata*, and *Eupenicillium hirayamae*. Sangale et al. performed a similar study on LDPE in twelve different eco-geographic sites on the west coast of India. In total, one hundred and nine fungal isolates were collected [57]. The most effective fungal isolates in PE degradation were identified, using morphological and molecular tools, as *A. terreus* strain MANGF1/WL and *Aspergillus sydowii* strain PNPF15/TS. Paço et al. exposed PE pellets to *Zalerion maritimum* and the results showed that, under the tested conditions, *Z. maritimum* is able to utilize PE as a carbon source, resulting in a decrease in PE mass and pellet size [8]. *Penicillium simplicissimum* LAR 13 and *Paecilomyces farinosus* LAR 10 presented good degradation efficiency versus PHB [58]. The highest degradation rates of PHB were induced by *P. simplicissimum* LAR 13, while *A. fumigatus* LAR 9 shows good efficiency in degrading Mater-Bi. Sang et al. found a microbial consortium colonizing poly(3-hydroxybutyrate-co-3-hydroxyvalerate) (PHBV) film surface [59]. The microbial consortium included fungi (*Fusarium oxysporium*, *Paecilomyces lilacinus*, and *Paecilomyces farinosus*) that strongly induced PHBV degradation. Finally, Matavulj and Molitoris were identified on PHBV, among other fungal species, including *Asteromyces cruciatus*, *Candida guillermondii*, *Debaryomyces hansenii*, and *Nia vibri* [60].

**Table 2:** List of fungal species reported in the literature responsible for plastic degradation, types of plastics involved, type of degradation detected, and localization of the activity, according to literature references (reported in the last column).

Fungi	Plastic Type	Observation of Degradations	Localization	Reference
*Aspergillus tubingensis*, *Aspergillus flavus*	HDPE	Weight loss; FT-IR observation; morphological observation (SEM)	Mangrove	[47]
*Aspergillus 1997* sp., *Penicillium* sp.	LDPE	Weight loss; morphological observation (SEM); formation of carbon dioxyde	Red Seawater	[56]
*Aspergillus caespitosus*, *Phialophora alba*, *Paecilomyces variotii*, *Aspergillus terreus*, *Alternaria alternata*, *Eupenicillium hirayamae*	LDPE	Morphological observation (SEM)	Red Seawater	[48]
*Aspergillus terreus*, *Aspergillus sydowii*	LDPE, PE	Weight loss; FT-IR observation; morphological observation (SEM)	Seawater and mangrove	[57]
*Zalerion maritimum*	PET	Weight loss	Seawater	[8]
*Penicillium simplicissimum*, *Paecilomyces farinosus*, *Aspergillus fumigatus*.	PHB	Weight loss; morphological observation (SEM)	Soils	[58]
*Asteromyces cruciatus*, *Candida guillermondii*, *Debaryomyces hansenii*, *Nia vibrissa*	poly-3-hydroxyalkanoates (PHA), PHB	Complete degradation	Seawater	[60]
*Fusarium oxysporium*, *Paecilomyces lilacinus*, *Paecilomyces farinosus*	PHBV	Weight loss; morphological observation	Soils	[59]

## 4. Degradation by Microalgae

Microalgae are ubiquitous microscopic photosynthetic organisms found both in marine and freshwater environments [61]. The degradation mechanisms of certain pollutants by microalgae are relatively well known, but little literature is available on the degradation of plastic materials by microalgae. The set of algal species whose ability to degrade plastic has been demonstrated in the literature as reported in Table 3. As for bacteria, the process usually starts with the colonization of the polymer surface. This is part of the marine fouling and affects the strength and physical performance of immersed polymers [62]. The hydrophobicity of the plastic subtract could limit the colonization process by algae [63]. Vimal Kumar et al. evaluated that, after a few weeks of exposure of the LDPE and HDPE films to the microalgae, the PE films were colonized by the microalgae, and erosion and degradation could be observed [64]. In addition, microalgae proliferated more on the LDPE films. Two pathways were identified during degradation by algae: (i) polymer molecular weight reduction, which is the degradation of large molecules initiated by enzymatic action; (ii) oxidation of low-molecular-weight molecules [65]. Sarmah and Rout evaluated the presence of enzymes interacting with available macromolecules (carbonyl groups) on the surface of PE, triggering biodegradation [63]. Later, it was shown that the functional expression of PETase in *Clamidomonas reinhardtii* was directly involved in polyethylene degradation [58]. The green alga *Chlorella fusca* was studied on bisphenol A (BPA) which is an endocrine disruptor when released into the environment [66]. It was able to remove almost all BPA in the concentration range of 10–80 mM for 168 h under continuous lighting at 18 W/m^2^. BPA was finally degraded to compounds with non-estrogenic activity.

Gulnaz and Dincer also conducted a study on BPA [67]. The results of the biodegradation test showed that bisphenol A BPA was readily biodegraded by *Chlorella vulgaris* at concentrations of 20 mg L^−1^ within 7 days. For PE, Sarmah and Rout highlighted the biochemical composition of five cyanobacteria isolated from the PE surface: *Phormidium lucidum*, *Oscillatoria subbrevis*, *Lyngbya diguetii*, *Nostoc carneum*, and *Cylindrospermum muscicola* [68]. Significant differences were shown between the biochemical parameters of cyanobacteria isolated from the PE surface. A total of 36 algae were found, colonizing the surface of PE carrier bags [68]. The most common species found were: (cyanobacteria) *Oscillatoria* sp., *Phormidium* sp., *Lyngbya* sp., *Nostoc* sp., *Arthrospira platensis*, *Hydrocoleum* sp.; (green algae) *Chlorella* sp., *Pithophora* sp., *Stigeoclonium tenue*; and (Diatoms) *Anomoeoneis* sp. *and Nitzchia* sp. Sharma et al. were also able to observe several algal species belonging to different genera on the degradation of PE: *Phormidium tenue*, *Oscillatoria tenuis*, *Monoraphidium contortum*, *Microcystis aeruginosa*, *Closterium constatum*, *Chlorella vulgaris*, and *Amphora ovalis* [69].

A study conducted on the biodegradation of LDPE by the microalgae *Uronema africanum* showed the degradation start after 30 days of incubation [70]. Sarmah and Rout also identified species colonizing and degrading PE [71]. In total, 20 species of the *Oscillatoria* genus were found. *Oscillatoria princeps*, *Oscillatoria subbrevis*, *Oscillatoria limosa*, *Oscillatoria amoena*, *Oscillatoria vizagapatensis*, *Oscillatoria okeni*, and *Oscillatoria laete-virens* are the most common species. Bhuyar et al. observed a consortium of *Chlorella* sp. and *Cyanobacteria* on PE samples collected in Malaysia, which was able to degrade LDPE sample through several steps, which was confirmed by the observation of a decrease in tensile strength and formation of low molecular weight compounds, but also by the observation of signs of alteration on the surface of the plastic [72].

Studies conducted on PET and PP have shown that the interaction between microplastics and microalgae *Spirulina* sp. is important [73]. *Spirulina* sp. was tested on PP and PET for 112 days and the results showed that the tensile strength of the PET decreased by 0.9939 MPa/day while the PP decreased by 0.1977 MPa/day, showing a role in the degradation process of the plastic. Similarly, Moog et al. created a consortium between the bacterium *Ideonella sakaiensis* and the alga *Phaeodactylum tricornutum* to generate a “cellular factory” capable of producing and secreting a modified version of PETase [74]. The results showed that PETase produced by diatoms was active against industrially shredded PET in a saltwater environment and demonstrated, through synthetic biology, which the diatom *P. tricornutum* can be converted into a valuable *chassis* for the biological degradation of PET.

**Table 3:** List of algal species reported in the literature responsible for plastic degradation, types of plastics involved, type of degradation detected, and localization of the activity, according to literature references (reported in the last column).

Algae	Plastic Type	Observation of Degradations	Localization	Reference
*Chlorella fusca*	Bisphenol A (BPA)	Weight loss	Aquatic and soil environment	[66]
*Chlorella vulgaris*	BPA	Decrease in concentration (Thermo Trace GC and GC-MS)	Algae culture laboratory	[67]
*Phormidium lucidum*, *Oscillatoria subbrevis*, *Lyngbya diguetii*, *Nostoc carneum*, and *Cylindrospermum muscicola*	PE	Growth of the alga using PE	Domestic sewage water	[75]
*Oscillatoria. limnetica, Phormidium calcicola*, *Oscillatoria earlei*, *Lyngbya cinerascens*, *Nostoc linckia*, *Spirulina major*, *Hydrocoleum* sp., *Pithophora* sp., *Scenedesmus quadricauda*, *Calothrix fusca*, *Stigeoclonium tenue*, *Calothrix marchica*, *Anomoeoneis* sp., *Oedogonium* sp., *Arthrospira platensis*, *Navicula minuta, Nitzschia* sp., *Navicula dicephala*, *Nitzschia intermedia*, *Spirogyra* sp. and *Synedra tabulata*	PE	Morphological observation	River	[68]
*Phormidium tenue*, *Oscillatoria tenuis*, *Monoraphidium contortum*, *Microcystis aeruginosa*, *Closterium constatum*, *Chlorella vulgaris*, and *Amphora ovalis*	PE	Morphological observation	Various ponds, lakes, and water bodies of Kota city	[69]
*Oscillatoria princeps*, *O. acuminate, O. willei*, *O. amoena*, *O. splendida*, *O. vizagapatensis*, *O. peronata*, *O. formosa*, *O. okeni*, *O. geitleriana*, *O. limosa*, *O. chalybea*, *O. salina*, *O. rubescens*, *O.curviceps*, *O. tenuis* and *O. laete-virens*	PET	Morphological observation	Domestic sewage	[71]
*Chlorella* sp. and *Cyanobacteria* sp.	PET	FT-IR observation; DSC	Seawater	[72]
*Spirulina* sp.	Polyethylene Terephthalate (PETE), PP	tensile strength; FT-IR observation; Morphological observation (SEM).	Freshwater	[73]
*Phaeodactylum tricornutum*	PETE	Ultra-high performance liquid chromatography (UHPLC); morphological observation (SEM).	Seawater	[74]
*Uronema africanum*	LDPE	Optical microscopy; dark field microscopy; gas chromatography; weight loss; FT-IR observation; SEM; atomic force microscopical (AFM).	Seawater	[70]

## 5. Influence of Environmental Factors on Degradation

Environmental conditions strongly influence the degradation behavior and rate of plastics. Environmental parameters such as moisture/water content, pH value, temperature, availability of oxygen and/or nutrients, oxidative and photo-oxidative environment, etc., affect polymer degradation. The complexity and variability of environmental parameters hinder a comparison between degradation processes in different compartments [76]. An attempt to compare polymer degradation in a different environment from soil biodegradation or composting and a marine environment is proposed to highlight material properties that hinder materials to degrade under marine conditions.

Polymer degradation in the soil is affected by soil texture, which is responsible for gas diffusion and water permeability, by precipitation, temperatures, pH values, and microorganisms [76]. The main standard test methods to measure the biodegradation of plastics in soil determine the biodegradability of plastics in soil under optimal controlled conditions favorable to the growth of mesophilic soil microorganisms [77]. Soil simulation studies, without following the specific standard tests, are also used to establish the material behavior under realistic exposure scenarios. Soil moisture and temperature are responsible for the hydrolytic cleavage of microorganisms, while pH affects the hydrolytic reaction and the rate of microbial growth [78].

Composting is described as a better-controlled process in terms of composition, pH, humidity, and size than biodegradation in soil [79].

Composting tests utilize biomass, lower test material concentrations, longer test durations, and elevated temperatures, typically 50–60 °C in industrial composting, a humidity of 45–60%, and a pH value between 6.5 and 8.0. There are different test guidelines that can be used to design compost studies describing many of the operational parameters, including test material-to-compost ratio, moisture content, and temperature. Moreover, there were several non-standard tests as well [78].

The degradation of PE samples induced by microorganisms has been tested in different environments. The durability of PE is mainly due to the presence of stable bonds as well as the absence of reactive functional groups, hydrophobicity, degree of crystallinity, and high molecular weight [15]. As a matter of fact, partial degradation and negligible weight loss were observed for PE in moist soil for 12–32 years [80]. Heat and ultra-violet light treatments of PE were identified to be essential to oxidize PE, reduce the molecular weight or increase it by crosslinking reactions, and modify the crystalline structure [81].

The degradation of two types of polymers is considered in this section, such as polymers with a carbon–carbon backbone and polyolefin and plastics with heteroatoms in the main chain, such as polyesters.

PE was susceptible to microbial colonization in soil [82]. Microorganisms have been found to affect PE, especially when the polymer is pretreated before being exposed to biological degradation. Microbial degradation tests allowed the isolation of various microorganisms from different types of soil (garden soil, forest soil, garbage soil, mangrove soil, and soil containing agricultural PE films for soil mulching) [83]. Moreover, PE degradation was studied using pure cultures or complex microbial communities from various terrestrial (soil from landfill sites and composting) and marine habitats [83,84].

**Figure 2:**
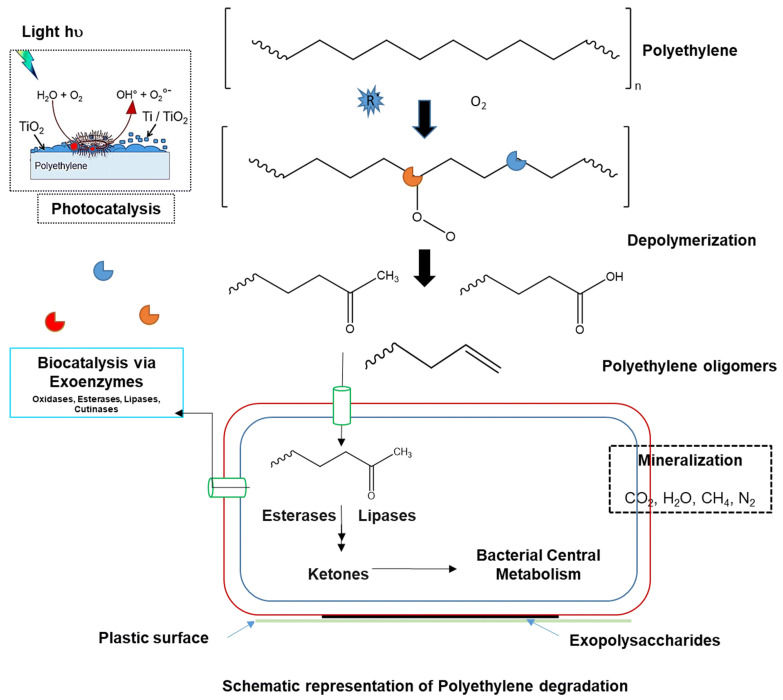
Abiotic and biotic factors could affect degradation of PE in the environment [84]. Copyright © 2020, Sunil Ghatge, Youri Yang, Jae-Hyung Ahn, and Hor-Gil Hur. Open Access licensed under a Creative Commons Attribution 4.0 International License which permits unrestricted use, distribution, and reproduction in any medium, provided the original work is properly cited.

It was suggested that different abiotic and biotic factors could affect the degradation of PE in the environment as schematized in Figure 2. Abiotic oxidation of PE, due to light exposure, is the initial degradation step in the environment that is followed by a propagation phase, radical reactions take place, with the formation of low molecular weight fragments, such as aliphatic carboxylic acids, alcohols, aldehydes, and ketones. Microorganisms can further degrade PE [85].

The formation of oxidized groups on PE chains results in increased hydrophilicity and a better attachment of microbes to the PE surface leading to biodegradation [86].

Laccase, manganese, and lignin peroxidases, enzymes able to degrade lignin polymer containing oxidizable C–C bonds, have been identified to degrade PE [87].

The biotic and abiotic processes for PE degradation can occur in all environments on different time scales. In the marine environment, the degradation processes will be even slower, as the conditions (lower temperature, lower oxygen, not complete light exposure, salinity), are not favorable for polymer degradation.

Chamas et al. harmonized polymer degradation data, reported in the literature, by calculating the specific surface degradation rate (SSDR), with the aim to estimate the half-life of different polymers in different environments. SSDR of LDPE with a typical thickness of 100 µm was esteemed to be 11 μm year^−1^ for polymer buried in the land, 22 μm year^−1^ (min 1.68; max 83) for degradation in land accelerated by UV or heat, 15 μm year^−1^ (min 0; max 37) for degradation in marine, and 10 μm year^−1^ (min 9; max 12) for degradation in marine accelerated by UV and/or heat [15].

Environmental factors affect the degradation rate, but this also depends strongly on the type of plastic. Environmental factors such as temperatures, oxygen, UV radiation, and biofilm formation can also vary in the same environment. In fact, some studies indicate that polymer degradation rates are lower in the marine environment compared to landfills due to lower temperatures and oxygen concentrations in the marine environment [88]. Other studies suggest that more intense UV radiation in a marine environment, relative to a landfill, could enhance degradation. In other landfills and industrial composters, the temperature can reach 80–100 °C, thus allowing, in an aerobic environment, the thermal-oxidative degradation and hydrolysis pathways [89]. Polymers buried in landfill, soil, and compost environments are not affected by solar UV radiation and a similar hindering can also be obtained in a marine environment as a consequence of biofouling [90].

Considering PLA, several studies reported PLA biodegradation [91]. The PLA degradation process occurs in two steps: (1) chemical hydrolysis and (2) microbial degradation with the formation of carbon dioxide under aerobic conditions and methane under anaerobic conditions [92]. Temperature strongly affects the PLA degradation rate that occurs fast under thermophilic (>58 °C) aerobic and anaerobic conditions while it occurs slowly around ambient temperature [93]. Recently, the degradation of PLA was evaluated in aerobic and anaerobic, aquatic, and solid-state conditions. Results highlighted a clear effect of temperature on the biodegradability of PLA in aerobic aquatic tests, as well as in an anaerobic solid state, suggesting that PLA should be hydrolyzed before microorganisms can utilize it as a nutrient source. A similar trend was observed in the natural composting process [91]. The degradation rate of PLA is low in soil and marine environments because the Tg of PLA is typically higher than the water temperature of the environment. Considering the environmental factors, soil moisture, temperature, and pH affect the hydrolytic reaction and the rate of microbial growth and, therefore, the rate of degradation [94]. As a matter of fact, the same polymer can present different rates of biodegradation in different environments. PLA did not hydrolyze in marine environments, where the temperature rarely reaches 30 °C and the main degradation process is due to photo-oxidation [95]. PLA complete degradation was reported to take about 1 year in soil, and from 60 to 100 days in compost [96]. Moreover, a slight weight loss was recorded for PLA buried in the sand after 9 months [97]. Martin et al. observed no weight loss after 45 days in seawater [98]. These data, in line with a previous review, suggest the promotion of the use of some biodegradable polymers in specific applications [99].

## 6. Conclusions and Future Perspectives

Oceans are highly affected by plastic pollution and the importance of studying the degradation of plastics by marine organisms is crucial. Although plastic products are a very useful tool for modern society, providing comfort and convenience at low cost in everyday life, the increasing amount of plastic waste is becoming a global concern, as it is greatly increasing environmental pollution. The review highlighted the biodegradation of different plastics due to three main marine organism groups: bacteria, algae, and fungi. Moreover, a wide range of enzymes used by microorganisms to achieve plastic degradation is reported. The improvement of knowledge of these enzymes and related activities could allow the development of bioremediation and biotechnology approaches. According to the literature, several enzymes seem promising for bioremediation purposes, such as laccase, peroxidase, cutinase, and esterase. However, plastic biodegradation is a slow process requiring a long time and, in this respect, the action of the enzymes needs to be studied and improved by the biotechnology approach. Many studies on the use of biotechnology to solve environmental pollution by plastics are emerging. In 2018, Austin et al. exchanged two active sites in the cutinase in order to conserve amino acids and thus increase PET degradation [100]. This opened the way for the subsequent combination of two enzymes and showed better efficiency than when the enzymes acted alone [101]. Studies have shown that the yield when combining enzymes can be increased eight times and up to forty times for PET [102,103]. The identification of optimal degradation conditions, efficient consortium, and of the use of several bacterial, algal, or fungal species, will strongly contribute to the development of bioremediation approaches for plastics. Many bioremediation pathways are being investigated and the use of marine microorganisms may present a real opportunity for these applications and increase the panel of organisms capable of degrading plastics. 

## Data Availability

Not applicable.
